# Host Disease Tolerance Predicts Transmission Probability for a Songbird Pathogen

**DOI:** 10.1002/ece3.70882

**Published:** 2025-03-12

**Authors:** Amberleigh E. Henschen, Francis E. Tillman, Sarah Coleman Ruston, Dana M. Hawley, James S. Adelman

**Affiliations:** ^1^ Department of Biological Sciences University of Memphis Memphis Tennessee USA; ^2^ Department of Biological Sciences Eastern Illinois University Charleston IL USA; ^3^ Department of Biological Sciences Virginia Tech Blacksburg Virginia USA

**Keywords:** bird, disease tolerance, host competence, house finch, *Mycoplasma gallisepticum*, pathogen transmission

## Abstract

Disease tolerance reduces the per‐pathogen fitness costs of infection for hosts and is an important component of host adaptation to pathogens. However, how disease tolerance affects host transmission potential is not well understood, especially because there are many potential mechanisms that facilitate host tolerance. For example, tissue‐specific host tolerance leads to the reduction of host pathology, regardless of pathogen load. Hosts may also exhibit behavioral tolerance, where normal behaviors are maintained even while harboring high pathogen loads. Here, we examined the impacts that tissue‐specific and behavioral tolerance have on transmission in house finches ( *Haemorhous mexicanus* ) infected with a common and highly transmissible bacterial pathogen, *Mycoplasma gallisepticum* (MG). MG causes conjunctivitis in house finches and severely reduces population numbers after it arrives in a new area. Wild house finch populations differ in tissue‐specific tolerance to MG and here we assessed how this variation in tolerance influences transmission success. We inoculated wild‐captured, MG‐naïve individuals from two populations that are on the extremes of tissue‐specific tolerance to MG and determined the likelihood of these “index” individuals transmitting MG to an uninfected, susceptible cagemate. Higher tissue‐specific tolerance results in reduced conjunctivitis, which is associated with decreased deposition and spread of MG. Thus, we predicted that individuals with high tissue‐specific tolerance would be less likely to transmit MG. In contrast, we predicted that behavioral tolerance would be linked to higher transmission, as more tolerant individuals spent more time on a feeder shared with a susceptible individual despite high pathogen loads. In agreement with our prediction, individuals with high tissue‐specific tolerance were less likely to transmit MG. However, there was no effect of behavioral tolerance on the likelihood of MG transmission. Our results highlight that it is key to consider how different mechanisms of tolerance affect transmission and, therefore, host‐pathogen coevolution and epidemic dynamics.

## Graphical abstract

The effects of host disease tolerance on pathogen transmission are not well understood. Here, we use wild‐caught house finches to show that natural variation in tissue‐specific tolerance to a bacterial pathogen affects the transmission of this pathogen. In contrast, host behavioral tolerance does not affect the transmission of the same bacterial pathogen in house finches.

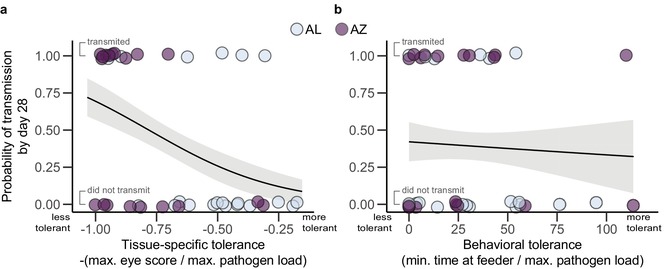

## 1 Introduction

Disease tolerance decreases the fitness costs of infection in hosts without decreasing pathogen load (Råberg, Sim, and Read 2007; Read, Graham, and Råberg 2008; Råberg 2014). Tolerance is an important mechanism for host adaptation to emerging pathogens in a wide range of wild taxa (Savage and Zamudio 2016; Bonneaud et al. 2019; Guito et al. 2021; Weber et al. 2022; Henschen et al. 2023). However, the effect of disease tolerance on a host's ability to transmit pathogens (i.e., host competence, Martin et al. 2016) is less well‐understood, and likely depends on the underlying mechanisms of tolerance and modes of pathogen transmission in a given host‐pathogen system (Adelman and Hawley 2017; Henschen and Adelman 2019; Seal, Dharmarajan, and Khan 2021).

In animals, disease tolerance can be broadly categorized as either tissue‐specific or behavioral, which can have opposite effects on host competence (Adelman and Hawley 2017). Tissue‐specific tolerance describes a variety of mechanisms that minimize pathogen‐associated damage to a host's own tissues or facilitate tissue repair (Medzhitov, Schneider, and Soares 2012; Adelman and Hawley 2017). In some cases, tissue‐specific mechanisms of tolerance are expected to increase host infectiousness, because tolerant hosts can harbor higher pathogen loads with reduced costs of infection, such as pathology and resulting infection‐induced mortality. In contrast, if the same pathologies that reduce host fitness also facilitate pathogen transmission (e.g., Hawley et al. 2023) tissue‐specific tolerance might instead inhibit pathogen shedding (Leggett et al. 2017). Thus, tissue‐specific tolerance may increase or decrease host competence, depending on the mechanisms of disease tolerance for a given system (Henschen and Adelman 2019). Behavioral tolerance refers to mechanisms that allow maintenance (or potentially augmentation) of fitness‐enhancing behaviors during infection, such as foraging and reproductive behaviors, and the suppression of sickness behaviors (Medzhitov, Schneider, and Soares 2012; Adelman and Hawley 2017; Stephenson and Adelman 2022), such as lethargy and anorexia (Tizard 2008; Ashley et al. 2011). In many host–parasite systems, the maintenance of normal behaviors in tolerant individuals likely increases host competence through increased contact rates with other susceptible hosts (Boots et al. 2009; Adelman et al. 2015; Burgan et al. 2019; Langager, Adelman, and Hawley 2023).

House finches ( *Haemorhous mexicanus* ) provide an excellent opportunity to study the links between tolerance and transmission, as these birds vary in their expression of both tissue‐specific and behavioral tolerance to the recently emerged bacterial pathogen *Mycoplasma gallisepticum* (MG) (Adelman, Kirkpatrick, et al. 2013; Bonneaud et al. 2019; Ruden and Adelman 2021; Henschen et al. 2023). In house finches, clinical signs of MG infection include lethargy, anorexia, and severe conjunctivitis (Ley, Berkhoff, and McLaren 1996; Luttrell et al. 1998) and mortality is likely indirect, caused by factors such as reduced predator avoidance (Adelman, Mayer, and Hawley 2017). Bird feeders act as important fomites for MG in wild house finch populations (Dhondt et al. 2007; Adelman et al. 2015). Tissue‐specific tolerance to MG, measured as maximum conjunctivitis versus maximum pathogen load, varies between populations of house finches: populations where MG has been endemic for  > 20 years (eastern United States) have evolved higher tissue‐specific tolerance to infection with MG compared to house finch populations where MG has been endemic for 0–15 years (western United States) (Henschen et al. 2023). More severe conjunctivitis during infection with MG increases the relative amount of conjunctival pathogen deposition onto bird feeders (Adelman, Carter, et al. 2013; Adelman and Hawley 2017), as well as the spread of inert fluorescent powder from an infected bird's conjunctiva to flockmates (Hawley et al. 2023), and is positively correlated with transmission (Williams et al. 2014; Bonneaud et al. 2020). Thus, by reducing the degree of per‐pathogen conjunctivitis, increased tissue‐specific tolerance in house finches likely decreases host competence to transmit MG. Behavioral tolerance to MG, measured as feeding frequency versus maximum pathogen load, has been shown to vary within a population. As house finches typically forage in flocks, the maintenance of normal feeding behaviors through tolerance likely increases contact rates among infected and uninfected individuals and, thus, transmission (Ruden and Adelman 2021). A prior study of a single eastern house finch population does indeed suggest that increased tissue‐specific tolerance reduces MG transmission and increased behavioral tolerance increases MG transmission (Ruden and Adelman 2021).

Here, we expand on previous work by determining the effects of individual and population‐level variation in tissue‐specific and behavioral tolerance on MG transmission success. We defined tissue‐specific tolerance by the relationship between maximum conjunctivitis and maximum pathogen load. We defined behavioral tolerance by the relationship between minimum feeding frequency and pathogen load because sharing feeders is a common transmission pathway in this system (Dhondt et al. 2007). We use individuals captured from two populations across the host's geographic range that differ greatly in the time of MG endemism ( < 10 years vs.  > 20 years), and thus tissue‐specific tolerance (Henschen et al. 2023). By using birds from these two populations, we capture a larger range of natural tissue‐specific (and potentially behavioral) tolerance than would be found within a single population (Henschen et al. 2023), yielding a more powerful design to detect effects on transmission. We performed experimental transmission studies in a common, captive environment using MG‐naïve individuals captured from these populations. Pairs consisted of one experimentally infected bird (=index birds) from either the more or less tolerant population and another, uninfected “cagemate” from a third population. Using cagemates captured from a third population with intermediate tissue‐specific tolerance ensured that pathogen transmission probability was not confounded by potential population differences in behavior or susceptibility among the uninfected cagemates. We determined whether index birds from our focal populations transmitted MG to a non‐infected, susceptible cagemate within 28 days. We predicted that (1) higher tissue‐specific tolerance in index birds would decrease the likelihood of MG transmission, (2) higher behavioral tolerance in index birds would increase the likelihood of MG transmission, and (3) tissue‐specific and behavioral tolerance would be positively correlated, decreasing the overall impact that differences in tolerance have on transmission in this system.

## 2 Materials and Methods

### 2.1 Ethics Statement

All collection and experimental methods in this study were approved by the University of Memphis (UM) IACUC, the State of California Department of Fish and Wildlife (permit #S‐190290001‐19044‐001), the State of Arizona Game and Fish Department (permit #SP406779), the State of Alabama Department of Conservation and Natural Resources (permit #10005), the State of Tennessee Wildlife Resources Agency (permit #35257117), and the U.S. Fish and Wildlife Service (permit #MB82600B).

### 2.2 Capture and Populations

Prior results showed that finches from Alabama (AL) have higher tissue‐specific tolerance during MG infection than did finches from either Arizona (AZ) or California (CA) (Adelman, Kirkpatrick, et al. 2013; Bonneaud et al. 2019; Henschen et al. 2023). We captured juvenile (hatch‐year) house finches, aged by plumage characteristics (Pyle 1997), from July to August, 2021 in Davis, CA; Tempe, AZ; and Auburn, AL using feeder traps and mist nets. We immediately released birds that had any potential MG‐related pathology (i.e., conjunctivitis). After capture, we weighed birds to the nearest 0.1 g and banded each bird with a captive metal band marked with a unique number.

### 2.3 Experimental Design and Timeline

We paired each index bird from the more‐tolerant (AL; *n* = 20) or less‐tolerant (AZ; *n* = 20) populations with a cagemate from a third population (CA) with an intermediate level of tolerance. We chose to pair AL and AZ birds with birds from a third population to reduce the possibility that individuals would be more likely to associate with or transmit MG to birds from their own populations, and to control for other potential contributions of cagemate population origin in transmission probability. Pairs were housed together in flight cages and shared a single bird feeder. We banded all index birds (i.e., those from AL and AZ) with a white leg band and all cagemates (i.e., those from CA) with a blue leg band so pairs could be distinguished visually.

To allow sufficient time for sampling on each day, we split birds into two groups, with inoculations spaced 2 days apart. To allow time for birds to acclimate to a new cage and cagemate, we paired all birds 14 or 16 days before inoculations (for the first and second groups, respectively). Two days later, we placed tube feeders with only a single open feeding port in each cage (12 or 14 days before inoculations, respectively). We filled tube feeders daily and removed open‐top food dishes for an increasing number of hours each day to help birds gradually transition to tube feeders. We removed one pair of birds (one AZ and one CA bird) from the experiment before inoculation because they failed to transition to tube feeders.

On day 0 of the experiment (15 or 17 September, 2021, for the first and second groups, respectively), we inoculated all birds from AL or AZ (index birds) with an isolate of MG taken from wild house finches in 1994 (35 μL per eye of a solution containing 7.5 × 10^6^ color‐changing units per mL of 7994‐1 6P 1:100 9/17/18) (Ley, Berkhoff, and McLaren 1996). After we inoculated AZ and AL birds on day 0, we quantified pathology (conjunctivitis) and pathogen load (see details below) of both inoculated index birds (AL, AZ) and non‐inoculated cagemates (CA) over 28 days. We quantified pathology on day post infection (DPI) 2, 4, 7, 9, 11, 14, 16, 18, 21, 23, 25, and 28 and quantified pathogen load on DPI 4, 7, 11, 14, 18, 21, 25, and 28. We also quantified behavior (number of visits to the feeder) of index birds (AL and AZ birds) for a 30‐min period each week (details of behavioral recordings below). All birds were humanely euthanized after DPI 28.

### 2.4 Transportation and Housing

We transferred birds from the field into captivity within 45 min of capture. We temporarily housed birds for 2–8 days in individual (0.6 × 0.4 × 0.3 m) or flight cages (1.8 × 1.8 × 2.4 m) in AZ and CA, respectively. We then transferred birds to the University of Memphis (UM) in Memphis, TN by plane and car in International Air Transport Association (IATA)‐approved animal carriers modified for safe avian transport. We transferred birds from AL to UM the day of capture by car using flock cages (76 × 46 × 46 cm). During transportation and housing in AZ and CA, we gave birds ad libitum access to food (black oil sunflower seed) and water (fresh fruit was substituted for water during flights per IATA regulations).

After birds were transferred to the UM, we housed birds in flight cages either individually or in pairs. In the first weeks at UM, we gradually transitioned birds from 100% black oil sunflower seed to a 20:80 mixture of sunflower seed and pellets (Roudybush Maintenance Nibles; Roudybush Inc., Woodland, CA). We gave birds ad libitum access to water and kept temperature (~21°C) and light cycles (12 h light:12 h dark) constant.

### 2.5 Quarantine Procedures

After birds arrived at UM, we housed them individually or in pairs (with an individual from the same population) in flight cages before our captive experiments began. We held all birds in captivity for a minimum of 44 and maximum of 50 days before the start of our experiment (i.e., the day index birds were inoculated).

On days 3, 7 and 14 in captivity, we inspected all birds for signs of MG infection (i.e., conjunctivitis). We also collected a small blood sample (~75 μL) from each bird (14–21 days after their arrival in captivity) to test for anti‐MG (IgY) antibodies, which would indicate a previous exposure to MG. We collected blood from the brachial vein in heparinized capillary tubes and then transferred these samples into 0.5 mL tubes. We stored tubes on ice (~4 h) until centrifugation for 10 min to separate and collect the plasma. To detect anti‐MG antibodies, we used a kit (99‐09298, IDEXX, Westbrook, Maine) with modifications as previously described (Hawley et al. 2011; Adelman, Kirkpatrick, et al. 2013). As the birds used in this experiment were hatch‐year juveniles (likely  < 3 months old), this is a powerful way to screen out any individuals that have been previously infected with MG.

Because wild‐caught house finches are often infected with a number of other pathogens, we gave each bird prophylactic medications to prevent morbidity and mortality in captivity. Briefly, we treated birds with Cankerex (MedPet, Newport, WA, USA) to minimize the risk of trichomoniasis, Endocox (2.5% toltrazuril, Jedds Bird Supplies, Anaheim, CA, USA) to prevent coccidiosis, and chloroquine and primaquine to eliminate malarial parasites. We followed the same treatment doses and regimen as reported in Henschen et al. (2023).

### 2.6 Pathology (Conjunctivitis) Quantification

We quantified pathology (conjunctivitis) by assigning birds an “eye score” on a scale of 0–3 at 0.5 intervals, a technique modified from Sydenstricker et al. (2006) (Hawley et al. 2011; Henschen et al. 2023). An eye score of zero indicated a healthy eye, with no swelling or redness, a score of one indicated minor swelling of the conjunctiva and eye rim, a score of two indicated moderate swelling, and a score of three indicated severe swelling. We combined scores from the same sampling day for each eye, giving each bird a score of 0–6 on each sampling day. A single investigator (JSA), blind to population of origin, assigned all eye scores throughout the experiment to prevent inter‐observer inconsistencies.

### 2.7 Pathogen Load Quantification

After eye scores were recorded, we swabbed the lower inner eyelid of each bird to measure MG load using a small cotton swab that was first dipped in Tryptose Phosphate Broth (TPB). After exposing the interior portion of the lower eyelid with sterilized plastic forceps, we moved swabs back and forth along the inner eyelid five times, starting at the edge of the eyelid and rotating the swabs continuously. We then transferred each swab to a fresh 1.5 mL tube of 300 μL TPB and repeatedly (five times) immersed swabs in the buffer and then pressed swabs to the side of the tube to extract all liquid. These samples were stored on ice during sample collection (2–6 h) and then stored at −20°C until DNA extraction.

We extracted DNA from TPB samples using a DNeasy 96‐well Blood and Tissue kit (Qiagen, Valencia, CA). Briefly, we transferred samples to a 96‐well lysis plate (provided by the kit) and incubated samples with the provided lysis buffer at 56°C overnight (~16 h) on a rotating platform. We then transferred samples to the provided 96‐well extraction column plate and extracted samples as per kit instructions, eluting each sample in 100 μL of elution buffer.

After DNA extractions, we quantified the amount of MG DNA in each sample using a qPCR that targets the *mgc2* gene (Grodio et al. 2008). Each PCR reaction (15 μL total) contained 3.525 μL of DNase‐free water, 7.5 μL of PrimeTime Gene Expression Master Mix, 0.375 μL of forward and reverse primers, 0.225 μL of probe, and 3 μL of template DNA. We ran qCPR assays for 3 min. at 95°C followed by 40 cycles of 3 s. at 95°C and 30 s. at 60°C. Samples were compared against a standard curve consisting of 8 standards that ranged from 1.81E+01 to 1.81E+08 copies of the *mgc2* amplicon sequence (g‐Block, Integrated DNA Technologies, Coralville, IA, USA). We ran all standards in triplicate and samples singly. All values of maximum pathogen load, which were used to define tolerance (see Section 2.9), fell within our standard curve.

### 2.8 Quantifying Behavior

During the first 3 weeks following experimental inoculation, we captured video one time per week to quantify the interactions of index birds with the feeder, which we used to measure behavioral tolerance (see analysis below). Shortly after lights‐on at 07:00, cameras (GoPro Hero 8, GoPro, San Mateo, CA, United States and Yi Action Camera, Yi Technology, Pudong District, Shanghai, China) were placed onto pre‐aligned holders on single cages, with a clear view to the bird feeder and its perch, so individuals could be identified by their colored leg bands. All cameras were turned on within 15 min of entering the room. Because of limited cameras, not all birds were recorded on the same day each week, with some animals recorded on DPI 4, 11, and 18 and others recorded on DPI 7, 14, and 21. Because of camera failures, we did not capture video for every bird on every day, but we detected no systematic bias in which days were missing between populations.

To ensure that the analyzed behaviors were not responses to observer presence, we discarded the first 30 min of each video and performed focal behavioral sampling on minutes 30–60 of each video. We measured the total time index birds spent at the bird feeder (defined as within one body‐width of the feeder port, regardless of whether the bird was actively eating). This is a relevant behavioral measure for this study as the time that an MG‐infected bird spends at a bird feeder predicts the probability of transmission in the house finch‐MG system (Adelman et al. 2015). In addition, this behavioral measure takes into account the possibility of birds making direct contact with cagemates at feeders and birds feeding from nearby perches rather than from the feeder itself. Two observers who were blind to treatment and population encoded behavior in BORIS (Behavioral Observation Research Interactive Software, University of Torino, Torino, Italy) (Friard and Gamba 2016).

### 2.9 Data Analysis

We calculated tolerance metrics for index birds using only data collected early in infection, up to and including DPI 11 for behavior and conjunctivitis, as the majority of transmission events were detected within this time frame or shortly thereafter. However, including all data for each bird does not qualitatively change our results. For each bird, we calculated tissue‐specific tolerance as:

$$-\left(\frac{\text{maximum}\;\mathrm{eye}\;\text{score}}{\text{maximum}\;\log_{10}\left(\text{pathogen load}+1\right)}\right)$$

across all samples up to and including DPI 11, which included five measures of eye score (DPI 2, 4, 7, 9, and 11) and three measures of pathogen load (DPI 4, 7, and 11). Similar to Ruden and Adelman (2021), for each bird, we calculated behavioral tolerance as:

$$\frac{\text{minimum proportion of time}\;\mathrm{at}\;\text{feeder}}{\text{maximum}\;\log_{10}\left(\text{pathogen load}+1\right)}$$

across all samples up to and including DPI 11, which included a single sampling day for 19 birds (DPI 4 or 7), two sampling days for 13 birds (DPI 2 and 4 or DPI 4 and 11), and three sampling days for six birds (DPI 2, 4, and 11). Populations (AL vs. AZ) were split approximately evenly among those sampling distributions (sampled once: 10 AL, 9 AZ; sampled twice: 6 AL, 7 AZ; sampled three times: 3 AL, 3 AZ). One pair was not sampled before dpi 11 and thus removed from the analysis. Time spent at the bird feeder was expressed as a proportion of the length of video observed (30 min). As in Ruden and Adelman (2021), we use a negative for the expression of tissue‐specific tolerance because pathology increases during infection, while feeding decreases. This negative operator means that higher values in both metrics indicate higher tolerance. As such, parameter estimates and graphical representations are more comparable between the metrics.

All analyses were performed in R v 4.1.3 (R Core Team 2022). We tested for population differences in both tolerance metrics using general linear models, correlations between tolerance metrics within individuals using Spearman rank correlations, and relationships between tolerance and transmission success (0 or 1) among individuals using a generalized linear model with a binomial error distribution and tissue‐specific tolerance and behavioral tolerance metrics as predictors. Because our primary motivation was looking at effects of tolerance variation on transmission, we chose to use each metric of tolerance, rather than population of origin, as continuous predictors in models of transmission.

## 3 Results

**FIGURE 1:**
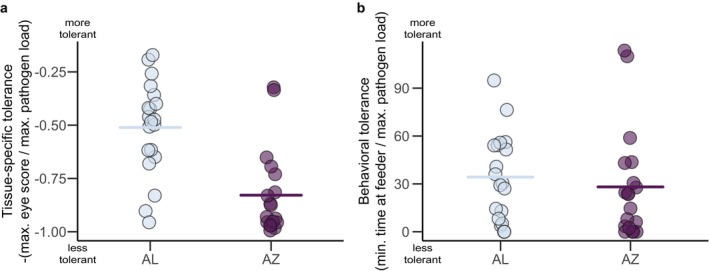
(a) Tissue‐specific tolerance was higher in Alabama (AL) than in Arizona (AZ) (linear model: Estimate (AZ) = −0.32, SE = 0.07, *t* = −4.71, *p* < 0.001) while (b) behavioral tolerance did not differ between these populations (linear model: Estimate (AZ) = −6.16, SE = 10.01, *t* = −0.62, *p* = 0.54).

**FIGURE 2:**
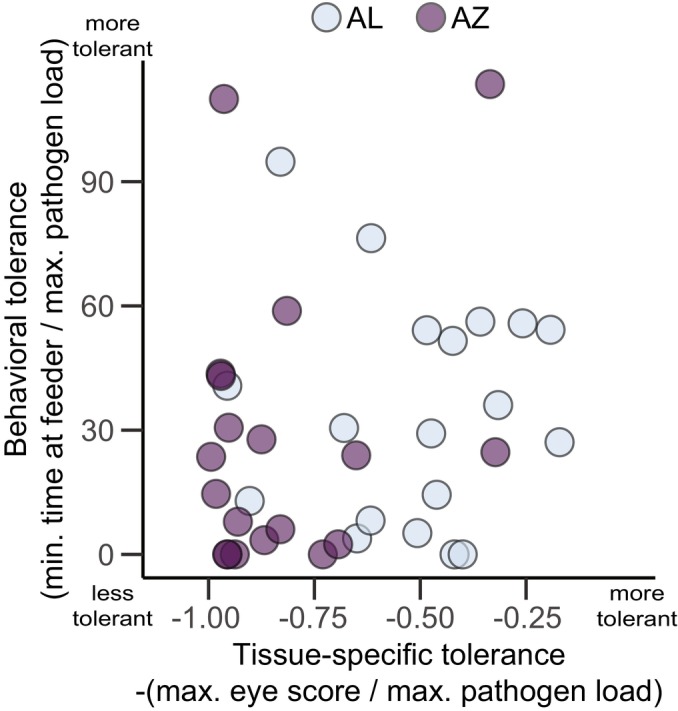
Tissue‐specific tolerance and behavioral tolerance in MG‐infected house finches were not correlated (*ρ* = 0.18, *S* = 7491.8, *p* = 0.28).

House finches from Arizona showed significantly lower tissue‐specific tolerance (higher peak pathology/peak pathogen load) during experimental MG infection than did finches from Alabama, although there was clear overlap in this metric between populations (Figure 1a, linear model: estimate (AZ) = −0.32, SE = 0.07, *t* = −4.71, *p* < 0.001). In contrast, index birds from the different populations showed no clear difference in average levels of behavioral tolerance, with considerable overlap in this metric between populations (Figure 1b, linear model: estimate (AZ) = −6.16, SE = 10.01, *t* = −0.62, *p* = 0.54). We detected no strong correlation between tissue‐specific tolerance and behavioral tolerance when individuals from both populations were analyzed together (Figure 2, *ρ* = 0.18, *S* = 7491.8, *p* = 0.28) or when individuals from each population were analyzed separately (AL: *ρ* = 0.07, *S* = 1063, *p* = 0.78; AZ: *ρ* = −0.01, *S* = 1157.1, *p* = 0.95).

**FIGURE 3:**
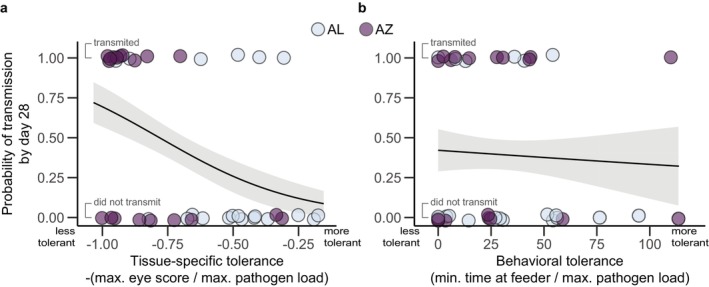
In our captive experiment, (a) higher tissue‐specific tolerance decreased the likelihood that a MG‐inoculated finch would transmit MG to a MG‐susceptible finch (logistic regression: Estimate = −3.75, SE = 1.56, *z* = −2.40, *p* = 0.016) while (b) behavioral tolerance did not have a detectable effect on the likelihood of MG transmission (logistic regression: Estimate = −3.8 × 10^−3^, SE = 0.01, *z* = −0.31, *p* = 0.76). Although index bird population of origin is labeled via color for visualization, population origin was not included in the statistical model because it is correlated with tissue‐specific tolerance.

We found a significant relationship between tissue‐specific tolerance of the index bird and transmission success to a cagemate, such that higher tissue‐specific tolerance (i.e., lower pathology/pathogen load) was associated with lower transmission (Figure 3a, logistic regression: estimate = −3.75, SE = 1.56, *z* = −2.40, *p* = 0.016). In contrast, we found no pronounced pattern between the probability of transmission and behavioral tolerance (Figure 3b, logistic regression: estimate = −3.8 × 10^−3^, SE = 0.01, *z* = −0.31, *p* = 0.76).

## 4 Discussion

Here we show that two distinct mechanisms of disease tolerance (tissue‐specific vs. behavioral) have different impacts on the transmission of a common bacterial pathogen (MG) of wild‐caught house finches. House finches with higher tissue‐specific tolerance, measured as decreased severity of conjunctivitis for a given pathogen load, were less likely to transmit MG to cagemates. In contrast, behavioral tolerance, measured here as increased time spent at a shared feeder for a given pathogen load, was not associated with the likelihood of transmitting MG.

Host disease tolerance is often hypothesized to increase pathogen transmission by reducing infection‐induced host morbidity or mortality (Martin et al. 2016; VanderWaal and Ezenwa 2016). In contrast, we predicted that tissue‐specific tolerance in the house finch‐MG system would be associated with lower transmission, because tolerance results in reduced conjunctival inflammation, a pathology that is closely linked to the relative amount of pathogen deposited on bird feeder surfaces (Adelman, Carter, et al. 2013) and transmission probability (Bonneaud et al. 2020). Indeed, we found that tissue‐specific tolerance was negatively associated with transmission probability, consistent with prior work in this system (Ruden and Adelman 2021). Although we found significant overlap between the range of tissue‐specific tolerance between index birds from Alabama and Arizona, on average birds from Alabama had higher tissue‐specific tolerance than birds from Arizona. This confirms previously reported differences in tissue‐specific tolerance in these populations (Henschen et al. 2023). Thus, using index birds from both Alabama and Arizona allowed us to get a larger sample size of birds from the extremes of the spectrum of tissue‐specific tolerance to MG in wild‐caught house finches.

While our results suggest that tissue‐specific tolerance is associated with lower transmission success, and thus pathogen fitness, a key caveat is that we measured transmission success in the absence of MG‐induced mortality. Because mortality from MG occurs indirectly for free‐living birds, due to factors such as predation of infected birds (Adelman, Mayer, and Hawley 2017) or inability to find food, it is largely absent in captive settings where birds are provided ad libitum access to food. Future work should use mathematical models to understand how predicted mortality during the early stages of infection might interact with tissue‐specific tolerance to influence overall transmission success in this system. In addition, we did not directly manipulate tolerance in this study so there is a possibility that the relationship between tolerance and transmission is driven by another confounding factor. Thus, it would be valuable to directly manipulate tolerance to MG in finches from different populations to determine if there is still a correlation between tissue‐specific tolerance and transmission probability.

Our tissue‐specific tolerance results suggest that it is important to understand how tissue‐specific pathologies are altered by tolerance to determine potential downstream effects on transmission (Henschen and Adelman 2019). For example, reduced tissue inflammation is a common hallmark of host tolerance to infection, and hosts in systems where tissue inflammation appears to increase pathogen shedding (Leggett et al. 2017; Zafar et al. 2017) may also evolve transmission‐reducing mechanisms of tolerance (Henschen and Adelman 2019). In contrast, reduced tissue inflammation in hosts during infection may increase pathogen transmission in systems where inflammation does not directly contribute to transmission (Burgan, Gervasi, and Martin 2018; Guito et al. 2021). Finally, it is possible that more than one type of host pathology is influenced by tolerance. Depending upon how each pathology relates to transmission, in such cases, it is possible that different mechanisms of tissue‐specific tolerance may impact transmission in synergistic or antagonistic ways.

We also examined effects of behavioral tolerance, which we predicted would covary with metrics of tissue‐specific tolerance. We chose a metric of behavior, and thus behavioral tolerance, that is directly relevant for MG transmission in the wild: time an infected bird spent on a feeder shared with a susceptible individual. This metric is particularly important in this system because bird feeders appear to act as important fomites (Dhondt et al. 2007), with birds who spend more time on feeders being both more likely to transmit and become infected (Adelman et al. 2015). Here we found significant variation in behavioral tolerance among MG‐infected house finches, even within populations. However, in contrast to previous work in this system (Ruden and Adelman 2021), we did not find any evidence to support our prediction that behavioral tolerance is associated with higher rates of transmission. The contrast between the two studies may be attributable to two main factors. First, we used slightly different protocols for measuring behavior (here, focal sampling; Ruden and Adelman 2021, scan sampling), although both metrics are likely to correlate positively. Second, here tolerance was calculated during infection as a fixed, early window, whereas Ruden and Adelman (2021) used a sliding window up until transmission. The use of a sliding window to measure behavioral tolerance results in individual birds contributing different amounts of behavioral data. Thus, the methods used in the current study are more conservative than those used in Ruden and Adelman (2021).

There are at least three additional considerations when interpreting the behavioral results of this study. First, we were able to quantify a single feeding behavior for only a short time during the experiment. It is possible that a longer sampling time, or a different behavioral measure, may reveal a pattern that we were unable to detect in this study. For example, the number of times a finch makes contact with the feeder, either with an infected eye or orally (as draining fluids from nasolacrimal ducts may also transfer pathogen), may more directly correlate with pathogen deposition on feeders. Second, to isolate potential patterns associated with host tolerance, our experiment was necessarily simple, with only a single MG‐inoculated and a single susceptible bird paired in a cage using the same feeder. In the wild, infected birds are likely to contact many susceptible hosts (either directly or through a fomite), which may increase the possibility of transmission. Indeed, in the wild, MG‐infected house finches prefer to forage near other house finches rather than alone (Langager, Adelman, and Hawley 2023). However, it is important to note that this simplified experimental design also applied to how tissue‐specific tolerance affects transmission, and we did detect an effect of that tolerance mechanism on transmission. Finally, in the wild or in larger flocks, infected finches may alter their behaviors at feeders, potentially leaving the feeder more than they would prefer to, due to competition. Predators may also result in wild finches leaving feeders more often than they would in a captive environment, although MG infection reduces anti‐predator behaviors in house finches (Adelman, Mayer, and Hawley 2017). Thus, heterospecific interactions might reduce transmission as they would result in finches remaining at feeders for a shorter amount of time. Finally, as noted above, effects of behavioral or tissue‐specific tolerance on mortality risk in the wild are important to consider, but could not be captured in our experimental design.

The lack of a correlation between tissue‐specific and behavioral tolerance in MG‐infected house finches was surprising. We found a full range of tolerance combinations, including individuals that had high behavioral tolerance and low tissue‐specific tolerance. Because these individuals spend the most time on the feeder and should shed the greatest amount of pathogen, they would be predicted to be superspreaders (Martin et al. 2019). However, because we found no evidence of a relationship between behavioral tolerance and transmission, these individuals did not transmit MG more often than individuals with low behavioral and low tissue‐specific tolerance. This may suggest that in this system, an individual that is more likely to deposit pathogen onto fomites is more likely to transmit, regardless of time spent in contact with fomites (although see Adelman et al. 2015). On the other hand, although we did see measurable variation in behavioral tolerance, it is possible the constrained spatial setting of a single feeder in a confined space swamped this behavioral variation, with all animals spending sufficient time on feeders to transmit, provided they shed sufficiently.

Overall, because we have evidence that tissue‐specific disease tolerance reduces MG transmission in house finches (Ruden and Adelman 2021, this paper), then the evolution of tolerance in this system (Henschen et al. 2023) may decrease pathogen fitness. Thus, tolerance would be predicted to affect host‐pathogen co‐evolution and epidemic dynamics in a manner similar to resistance (VanderWaal and Ezenwa 2016). Just as pathogens evolve to overcome resistance mechanisms that decrease their reproduction, and thus transmission potential, we would predict that pathogens would evolve mechanisms to counteract tolerance in this case. In the house finch‐MG system, we would predict that pathogens would evolve higher virulence to cause more severe conjunctivitis in house finch hosts. This prediction is consistent with the pattern of virulence evolution observed in this system, with more recent isolates showing higher virulence (i.e., inducing more severe conjunctivitis) (Hawley et al. 2013; Bonneaud et al. 2018; Tardy et al. 2019). Although other mechanisms underlying the evolution of increased virulence are also at play in this system, including partial immunity (Fleming‐Davies et al. 2018), these are certainly not mutually exclusive to a contribution from increased host tolerance. We also note that such a framework would complement prior theory suggesting that tolerance mitigates the costs that pathogens pay for virulence, thus selecting for increased virulence (Restif and Koella 2003, 2004; Miller, White, and Boots 2006; Carval and Ferriere 2010; Best, White, and Boots 2014). Given these potential links to pathogen evolution, future work in this and other systems should continue to explore the effects of disease tolerance by examining transmission‐relevant mechanisms of tolerance. In addition, experimental manipulation of co‐evolution, such as experiments that passage a pathogen through several generations or models, will be vitally important to predicting how tolerance alters the host‐pathogen dynamics in wildlife.

## Author Contributions

**Amberleigh E. Henschen:** conceptualization (equal), data curation (equal), formal analysis (equal), investigation (lead), methodology (equal), project administration (equal), supervision (equal), validation (equal), visualization (equal), writing – original draft (lead), writing – review and editing (equal). **Francis E. Tillman:** conceptualization (supporting), investigation (supporting), methodology (supporting), writing – review and editing (equal). **Sarah Coleman Ruston:** conceptualization (supporting), investigation (supporting), methodology (equal), writing – review and editing (equal). **Dana M. Hawley:** conceptualization (equal), funding acquisition (equal), methodology (equal), project administration (equal), resources (equal), supervision (equal), writing – review and editing (equal). **James S. Adelman:** conceptualization (equal), data curation (equal), formal analysis (equal), funding acquisition (equal), investigation (equal), methodology (equal), project administration (equal), resources (equal), supervision (equal), validation (equal), visualization (equal), writing – original draft (equal), writing – review and editing (equal).

## Conflicts of Interest

The authors declare no conflicts of interest.

## Data Availability

All data and code used in this study are accessible at: https://doi.org/10.5061/dryad.ttdz08m62.
